# Expression and Cellular Distribution of Ubiquitin in Response to Injury in the Developing Spinal Cord of *Monodelphis domestica*

**DOI:** 10.1371/journal.pone.0062120

**Published:** 2013-04-23

**Authors:** Natassya M. Noor, Kjeld Møllgård, Benjamin J. Wheaton, David L. Steer, Jessie S. Truettner, Katarzyna M. Dziegielewska, W. Dalton Dietrich, A. Ian Smith, Norman R. Saunders

**Affiliations:** 1 Department of Pharmacology, University of Melbourne, Parkville, Victoria, Australia; 2 Department of Cellular and Molecular Medicine, Faculty of Health Sciences, University of Copenhagen, Copenhagen, Denmark; 3 Department of Biochemistry and Molecular Biology, Monash University, Clayton, Victoria, Australia; 4 The Miami Project to Cure Paralysis, University of Miami, Miller School of Medicine, Miami, Florida, United States of America; Hertie Institute for Clinical Brain Research, University of Tuebingen, Germany

## Abstract

Ubiquitin, an 8.5 kDa protein associated with the proteasome degradation pathway has been recently identified as differentially expressed in segment of cord caudal to site of injury in developing spinal cord. Here we describe ubiquitin expression and cellular distribution in spinal cord up to postnatal day P35 in control opossums (*Monodelphis domestica*) and in response to complete spinal transection (T10) at P7, when axonal growth through site of injury occurs, and P28 when this is no longer possible. Cords were collected 1 or 7 days after injury, with age-matched controls and segments rostral to lesion were studied. Following spinal injury ubiquitin levels (western blotting) appeared reduced compared to controls especially one day after injury at P28. In contrast, after injury mRNA expression (qRT-PCR) was slightly increased at P7 but decreased at P28. Changes in isoelectric point of separated ubiquitin indicated possible post-translational modifications. Cellular distribution demonstrated a developmental shift between earliest (P8) and latest (P35) ages examined, from a predominantly cytoplasmic immunoreactivity to a nuclear expression; staining level and shift to nuclear staining was more pronounced following injury, except 7 days after transection at P28. After injury at P7 immunostaining increased in neurons and additionally in oligodendrocytes at P28. Mass spectrometry showed two ubiquitin bands; the heavier was identified as a fusion product, likely to be an ubiquitin precursor. Apparent changes in ubiquitin expression and cellular distribution in development and response to spinal injury suggest an intricate regulatory system that modulates these responses which, when better understood, may lead to potential therapeutic targets.

## Introduction

It is well established that the capacity of the central nervous system (CNS) to grow and repair itself after injury diminishes as it matures [1]. Although largely a mystery, evidence suggests that intrinsic molecular control affected by age is responsible for the reduced ability to repair and recover after trauma [2]. The most extensive developmental studies of mammalian spinal injury used two marsupial species of opossum: *Didelphis virginiana* [3] and *Monodelphis domestica* [4]–[5]. This is due to their physical immaturity at birth [6]–[7] and thus the ease of accessibility for experimental procedures, which in rodents and other eutherians would need to be carried out *in utero*. Use of *Monodelphis* as an animal model was further enhanced when its genome was published [8], making genome wide and transcriptomic studies possible.

We have demonstrated previously that following a complete spinal transection performed in the first week of life, the *Monodelphis* regenerate nearly 50% of axotomised neurites originating from supraspinal (brainstem) neurons [4]. These spinally transected animals demonstrated near normal locomotion when they have developed to adulthood [9]. However, if a similar injury is performed at four weeks of age, these animals do not re-grow new processes across the site of injury and their functional recovery is much diminished [9]. This raises the question of what has changed between these two stages of spinal cord development and what initiates the transition from intrinsic and environmental factors that are permissive for recovery (first week of life) to those that are non-permissive and no longer supportive of axon re-growth (four weeks of age).

Using a proteomic approach we have previously identified many proteins that changed differentially in response to spinal cord injury in the segment of the cord caudal to the lesion at these two developmental stages: the very immature postnatal (P) day 7 and a more developed P28 compared to their individual age-matched controls [10]. One of the identified proteins, ubiquitin, an ubiquitous protein known for its relation to the proteasome degradation pathway [11], showed a differential response to injury made at these two ages, as identified by a 2-D gel separation method and validated using western blotting and Reverse Transcription – quantitative polymerase chain reaction (qRT-PCR). When the cord was transected at P7 ubiquitin levels decreased whereas following transection at P28 ubiquitin increased [10]. We now extend this study to the rostral segment of the cord both during early development (P8 to P35) in *Monodelphis* and following a complete spinal transection. This has involved investigating changes in detectable levels of ubiquitin protein and its mRNA expression levels. We also include morphological studies of ubiquitin where we have identified cell populations in which ubiquitin immunoreactivity has been detected both in development and in response to spinal cord injury. Topographical areas of the spinal cord that showed changes in ubiquitin expression after spinal injury are also outlined.

## Methods

### Animals

#### Ethics Statement

Monodelphis domestica pups used in this study were obtained from a colony located at the Medical Sciences Animal House Facility at the University of Melbourne, Victoria, Australia. All surgical procedures conducted throughout this study conformed to the National Health and Medical Research Council (NHMRC) guidelines with ethics approval by the University of Melbourne Animal Ethics Committee (Ethics #1111998.1).

The day of birth is designated as postnatal day zero (P0). Animals of both sexes were used in this study. Pups were separated into two groups: (i) an experimental group where animals were subjected to a complete spinal cord transection at thoracic level 10 (T10), at either P7 or at P28 and were terminally anaesthetized one or seven days later (P7+1 d or P7+7 d and P28+1 d or P28+7 d) and (ii) age-matched controls (P8, P14, P29 and P35). In addition P21 pups were used in the profiling of ubiquitin for normal cord development. In this study, control groups were defined as animals that did not have any surgical procedures performed apart from undergoing anaesthesia for the same duration as experimental animals.

At P7 all pups in a litter were injured (n<10) as it is not possible to consistently mark them at this age without increasing the risk of cannibalization by their mother [9]. Separate litters were collected as age-matched controls. For the P28-injured group, approximately half of the pups (n<5) in the litter underwent the surgery whilst the other half were collected as age-matched controls.

#### Spinal cord transection

A complete spinal cord transection at T10 was performed as described in detail previously [4]–[5],[9]–[10]. Surgical procedures on P7 animals were conducted whilst the pups were still attached to their mother’s teats. The mothers were anaesthetized with 2–3% isofluorane and the same anaesthetic was also administered to the pups via a miniature facemask. P28 pups were individually anaesthetized throughout the procedure with 2–3% isofluorane.

Spinal cord injury was performed using sharp sterilized scissors (P7) or a sterilized surgical blade (P28). Mothers with their pups were returned to their cages post-surgery for one day (+1 d) or seven days (+7 d) recovery depending on the experimental group. At the end of the recovery period, pups were terminally anaesthetized with an overdose of isofluorane. Spinal cords were dissected out on ice and separated as cord segments upper (rostral) and lower (caudal) to the site of the injury. In this study only the upper spinal cord segments were used as we have already published a proteomic study of the lower segment in these same animals [10]. In order to increase the biological reproducibility of the results, different pups from several litters were pooled together in all three experimental procedures described in this paper: western blotting, qRT-PCR and proteomics. For histological examination, 3–4 spinal cords were used at each age.

#### Western blotting

Upper spinal cord samples were pooled from several pups (n = 2–10) to obtain a total wet tissue weight of 10–15 mg per sample. At all ages two separately pooled samples were analyzed to confirm the results. Cords were homogenized in 1∶10 weight/volume in a buffer containing 0.32 M sucrose, 25 mM Tris, 1 mM MgCl2, pH 7 using syringes with varying size needles (20, 21, 25 and 27 Gauge). Extracts were centrifuged and supernatants containing soluble proteins collected. Total protein quantification using the Bradford Assay was performed on all samples [12].

Lithium dodecyl sulfate – polyacrylamide gel electrophoresis (LDS-PAGE) was performed following the Manufacturer’s Protocol using pre-cast 4–12% NuPAGE Bis-Tris gels (Invitrogen) with 2-(Nmorpholino)ethanesulfonic acid (MES) running buffer at 200 V for 35 minutes as described in detail previously, as were the conditions for Western blotting protocol and analysis [10]. Briefly, separated proteins were transferred onto polyvinylidine fluoride (PVDF) membranes using an iBlot (Invitrogen) for dry transfer. Blocking solution made of 1∶1 Tris-buffered saline (TBS) and soymilk was used overnight at 4°C. Membranes were incubated with cross-reacting antibodies to ubiquitin (DakoCytomation, Denmark, Code No. Z 0458) diluted 1∶200, followed by swine anti-rabbit (DakoCytomation, Denmark, Code No. Z 0196, 1∶400) and rabbit PAP (Sigma-Aldrich, Code No. A 0545, 1∶400), for two hours each at room temperature. Between each antibody change, the membrane was washed with TBS until solution was clear. The membrane was developed with diaminobenzidine (DAB). The reaction was stopped by immersing the membranes in distilled water. Developed membranes were left to dry overnight. They were digitally scanned and densitometric analysis was performed using GeneTools V4.01.02 software (Syngene, Synoptics Ltd, Cambridge, England).

#### Proteomic analysis

Samples of the spinal cord segments rostral (upper) to the injury site or equivalent segments from age-matched controls were pooled from several pups (n = 4–20) to obtain a total wet tissue weight of about 30 mg per sample. 2-D separation protocol was performed as described previously [10]. Cords were homogenized 1∶10 weight/volume in a buffer containing 0.32 M sucrose, 25 mM Tris, 1 mM MgCl2, pH 7 using syringes with varying needle size (20, 21, 25 and 27 Gauge) centrifuged and supernatants collected. Total protein quantification using the Bradford Assay [12] was performed on all samples.

Contaminants from lysates were removed using a 2-D clean-up kit (GE Healthcare) as described by the Manufacturer’s Protocol. Samples were fractionated using the 3100 Off-gel Fractionator (Agilent) following the Manufacturer’s Protocol on 12 cm low-resolution immobilized pH gradient strips (pH3–10). Samples were separated into 12 wells numbered as Fractions 1–12 and stored at −20°C until further analysis.

Aliquots from these fractions (25 µl) were separated on LDS-PAGE following the Manufacturer’s Protocol. LDS-PAGE was run for 35 minutes (200 V) using pre-cast 4–12% NuPAGE Bis-Tris 12 well mini gels (Invitrogen) on MES-SDS running buffer (Invitrogen, Carlsbad, CA, USA) with a molecular weight marker Novex Sharp pre-stained standard (Invitrogen). Samples were run in the order P7+1 d, P8, P7+7 d, P14, P21, P28+1 d, P29, P28+7 d and P35. All gels were run in duplicate. Separated protein bands were visualized using silver staining as described by the Manufacturer’s Protocol (Silver Stain Plus Kit, BioRad). Ubiquitin was identified using mass spectrometry and western blotting on LDS-PAGE gels. Ubiquitin was only detected in Fractions 7 and 8 therefore only data from these two fractions are described.

#### Histology and Immunocytochemistry

At the end of the experimental time (1 or 7 days after injury) animals were terminally anaesthetized by isofluorane overdose and exsanguinated. Spinal cords were dissected out and the tissue segments centred on the injury site together with 5 mm above and 5 mm below (and a corresponding spinal segment from control animals) were immerse-fixed in Bouin’s fixative overnight at room temperature. This was followed by washes in 70% ethanol until clear of colour. Cords were embedded in paraffin wax and consecutive sections cut on a Leica Microtome (Leica Microsysteme Vertrieb Gmbh, Wetzlar, Germany) at 5 µm in a transverse plane. Ten sections were placed on each silanised glass slide. Routine haematoxylin and Eosin (H&E) staining was done on every tenth slide for general morphology [10]. Selected slides were stained with antibodies to ubiquitin as described below.

#### Ubiquitin immunohistochemistry

Paraffin embedded sections were de-paraffinised and rehydrated in xylene followed by a series of graded alcohols, treated with a 0.5% solution of hydrogen peroxide in methanol for 15 minutes to quench endogenous peroxidase, and then rinsed in TRIS buffered saline (TBS, 5 mM Tris-HCl, 146 mM NaCl, pH 7.6). Non-specific binding was inhibited by incubation for 30 minutes with blocking buffer (ChemMate antibody diluent S2022, DakoCytomation, Denmark) at room temperature. The sections were then incubated 2 hours at room temperature with a rabbit polyclonal antibody that cross-reacts with human ubiquitin (DakoCytomation, Denmark, Code No. Z 0458), at concentration 1∶20000 diluted in blocking buffer. The sections were washed with TBS and incubated for 30 minutes with peroxidase labeled polymer conjugated to goat anti-rabbit/mouse immunoglobulins (EnVisionTM+ System/HRP K5007, DakoCytomation, Denmark). The sections were again washed with TBS, followed by incubation for 6 min with 3,3′-diamino-benzidine chromogen solution. Positive staining was recognized as a brown colour. The sections were counterstained with Mayers hematoxylin and dehydrated in graded alcohols followed by xylene. Slides were mounted using DPX mounting media and a cover slip was placed to seal the section. Control sections in which the primary antibody was omitted, were included in each run and always appeared blank.

#### Quantitative reverse transcription polymerase chain reaction (qRT-PCR)

Total RNA was extracted from individual spinal cord segments using Qiagen RNeasy ® mini kit (Valencia, California, USA) following Manufacturer’s Protocol. cDNA was reversed transcribed from these samples using Multi-Scribe Reverse Transcriptase (Applied Biosystems, Foster City, California, USA). qRT-PCR was performed on these cDNAs using SYBR Green chemistry with Taqman DNA polymerase and gene specific PCR primers for ubiquitin (forward primer: 5′-GGTGGTGCCAAGAAGAGAAA and reverse primer: 5′-ATAAAGACCCCAGCACCACA) and the housekeeping gene used, cyclophilin (forward primer: 5′-TCCAAAGGCAGCAGAAAACT and reverse primer: 5′-AAAACTGGGAGCCATTTGTG) [10] using the Applied Biosystems 7300 Real-Time PCR System. All samples were run in triplicate. Threshold cycle (Ct) values were obtained from triplicates, averaged and normalized to the housekeeping genes used in this study (cyclophilin) obtaining the ΔCt values. Results were then normalized to the average values of control samples obtaining the ΔΔCt values. Finally, results were analysed using the 2−ΔΔCt method to obtain fold changes (FC). Six to nine individual cord segments were collected for each age group of animals and analyzed separately.

#### Statistical analysis

Statistical evaluations were performed on the qRT-PCR data using the Mann-Whitney U test (un-paired, two-tailed, nonparametric test) with Graphpad InStat (Ver. 5.0, San Diego, USA) using individual FC values obtained from control and injured samples. P-values ≤0.05 were considered significant.

## Results

Changes in the expression of ubiquitin, both at the gene and protein level, were investigated in the developing *Monodelphis* following spinal cord transection, together with age-matched controls in the segment of the spinal cord rostral to the site of injury at either one day or seven days after transection at two postnatal ages: P7 or P28.

Samples of cord tissue following a complete spinal transection performed at T10 were collected from 6–9 pups for qRT-PCR. These were analyzed individually. For the proteomic analysis the tissue was pooled from several pups (n = 4–20, see Methods) to obtain enough material for mass spectrometry (about 30 mg tissue weight per sample). The required amount of tissue was based on our previous proteomic profiling of the lower spinal cord of *Monodelphis* at similar developmental ages [10]. Quantification of total protein using Bradford assay [12] showed that concentration of protein extracted from the present samples was similar between each age-matched group (Table 1).

**Table 1:** Number of cords, pooled tissue weight and total protein concentration of each sample used in the proteomic analysis.

Age group	Number of cords	Tissue weight (mg)	Total protein concentration (µg/µl)
**P7+1** **d**	11	37.7	4.7
**P8**	11	27.8	5.14
**P7+7** **d**	10	49.9	7.99
**P14**	11	45.4	5.99
**P21**	7	89.9	6.74
**P28+1** **d**	4	66.5	6.97
**P29**	4	87	8.74
**P28+7** **d**	3	82.5	8.74
**P35**	4	97.9	9.22

+1 d and +7 d refer to days after injury at either P7 or P28. P8, P14, P29 and P35 are age-matched controls.

### Western Blotting

**Figure 1:**
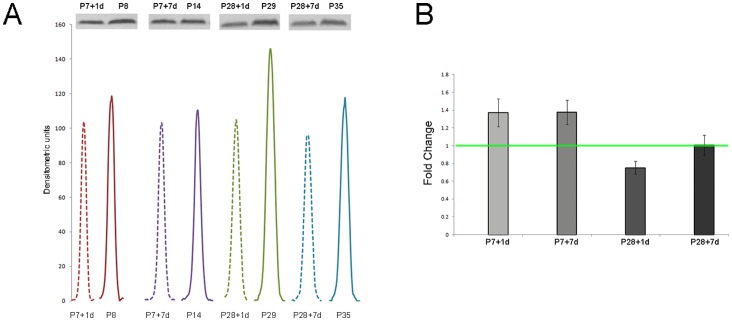
Western blot of ubiquitin, corresponding densitometry and qRT-PCR. (A) Western blot of spinal cord segment lysates (7 µl) separated on LDS PAGE and probed for Ubiquitin with corresponding densitometry graph. Reprication using independent samples gave similar results. (B) qRT-PCR of ubiquitin in the same spinal segment. Graph is shown as fold change of spinal cord injured animals compared to their age matched controls (taken as 1). N = 6–9 separate samples per age group. Level of significance was set as p<0.05.

Protein extracts of the spinal cord segments (pooled samples) from the injured animals (injury at postnatal day 7 or P28, collected one or seven days later, P7+1 d, P7+7 d; P28+1 d, P28+7 d) and age-matched controls (P8, P14, P29, P35) were separated by their molecular weight using 1-D polyacrylamide gel electrophoresis and transferred proteins were probed with cross-reacting antibodies to human ubiquitin [10]. Equal volumes were used for each sample. Results from western blots, including densitometry analysis, are presented in Figure 1A.

Between P8 and P35 in control cords levels of ubiquitin remained relatively similar except at P29 where they appear to be higher (compare peaks of solid lines in Figure 1A). Following spinal transection ubiquitin levels were mostly lower in all experimental age groups (dashed lines in Figure 1A), especially one day after injury at P28 (P28+1 d compared to P29).

Statistical analysis could not be performed on these results because western blots were run only on two separate samples. However both samples gave similar results as those illustrated in Figure 1A (see Limitations of the Study).

### qRT-PCR

In order to relate changes observed at the protein level to its mRNA expression, mRNA for ubiquitin was determined by qRT-PCR in the upper (rostral) spinal cord segment collected from different litters then those used for protein detection. In each age group 6–9 individual cord samples were analysed. Results are illustrated in Figure 1B as fold changes compared to control (2^−ΔΔCt^ see Methods). As can be seen in Figure 1B ubiquitin mRNA levels were apparently higher following injury at P7, lower one day after injury at P28 and not changed seven days after transection at P28. However these values only reach the level of significance (P<0.05) at P7+7 d.

### Proteomics

**Figure 2:**
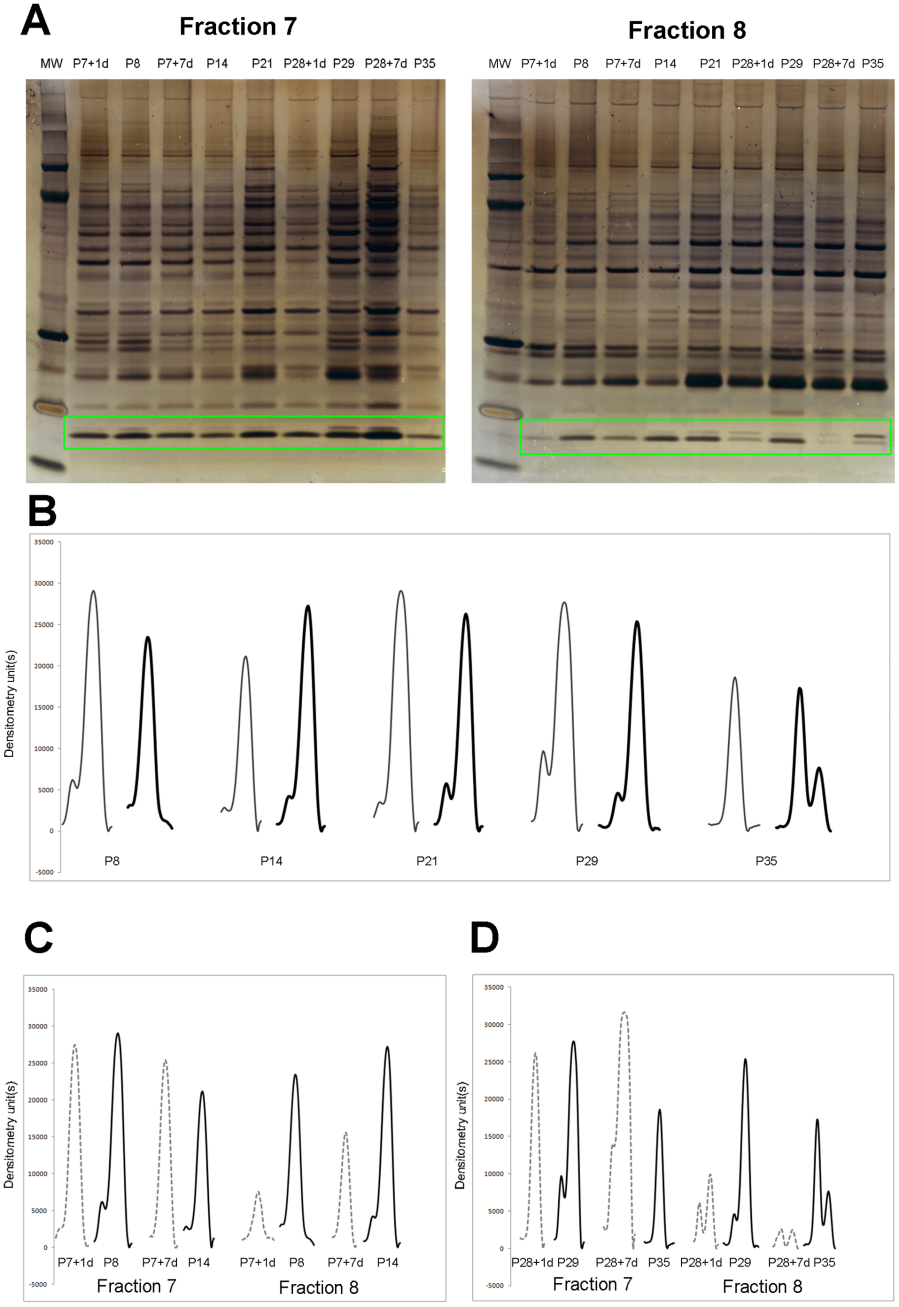
Silver stained gels and western blots for ubiquitin as identified using 2-D separation. (A) Silver stained gels of fraction 7 and 8 following separation by Off-gel electrophoresis and LDS PAGE. Ubiquitin was identified by mass spectrometry (boxed area) and also confirmed by western blotting (Figure 3). (B) Densitometry graphs of ubiquitin bands from silver stained gels from control animals only showing the protein during development. Lighter lines refer to Fraction 7 and darker lines refer to Fraction 8. (C) Densitometry graphs of ubiquitin bands from silver stained gels for P7 spinal cord injured animals (dotted lines) compared to age-matched controls (solid lines) in Fraction 7 and Fraction 8. (D) Densitometry graphs of ubiquitin bands from silver stained gels for P28 spinal cord injured animals (dotted lines) compared to age-matched controls (solid lines) in Fraction 7 and Fraction 8.

**Figure 3:**
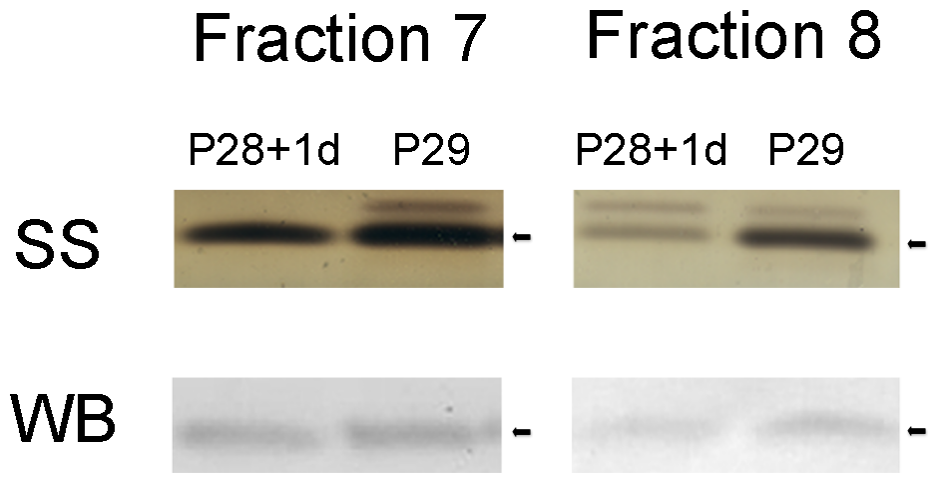
Silver stained gels and western blots of ubiquitin showing cross-reactivity of anti-ubiquitin antibody. Silver stained (SS) bands identified by mass spectrometry showing two protein bands. Higher band was identified as a fusion protein containing ubiquitin at the N-terminus and a ribosomal protein L40 at the C-terminus and the lower one was identified as mono-ubiquitin. Western blots (WB) of ubiquitin showing that the anti-ubiquitin antibody used only cross-reacts with a single band, as demonstrated in both Fractions 7 and 8 (black arrows).

In our previous proteomic studies of the spinal cord caudal to the lesion we have identified ubiquitin in Fraction 8 following gel separation using 2-D fractionation [10]. In the present study we have also identified ubiquitin in fraction 8, but in addition ubiquitin was also identified in fraction 7 (Figure 2A). In both fractions ubiquitin separated as two bands, which were more prominent at older ages (Figure 2A). Both bands in both fractions were identified as ubiquitin by mass spectrometry. However, only one of the bands, corresponding to the 8.5 kDa ubiquitin monomer, cross-reacted with the antibody, as shown previously [10]. The second, higher molecular weight band was identified as a fusion protein containing ubiquitin at the N-terminus and a ribosomal protein L40 at the C-terminus (a C-terminal extension protein) [13]–[14]. This fusion product did not show cross-reactivity with the ubiquitin antibody used as illustrated in Figure 3, which is a western blot of fractions 7 and 8. In both fractions the antibody only identified the lower molecular weight band.

Once ubiquitin bands were identified it was possible to analyze their developmental pattern in silver stained gels of fraction 7 and 8 (Figure 2A). Figure 2B is a densitometry analysis of ubiquitin bands during normal *Monodelphis* spinal cord development. Levels of ubiquitin present in fraction 7 (lighter line in Figure 2B) appeared different at different ages with lowest levels present at P14 and P35. On the other hand ubiquitin levels in fraction 8 (heavier band in figure 2B) seemed to increase between P8 and P14, remained steady until P29 and became lower at P35. However it should be noted that at P35 there was a significant shift between the two ubiquitin bands identified in silver stained gels: the upper band became more prominent than the lower band. At all other ages the upper band was only slightly visible as illustrated by a small peak detected by the densitometry scan. That peak was present on the left hand side of the main ubiquitin peak (marked with a star in Figure 2B).

Following spinal injury levels of ubiquitin seemed to change differentially in both fractions and between the two ages, as illustrated in Figure 2C and D. Transection at P7 (Figure 2C) resulted in only a small change in ubiquitin in fraction 7, but in fraction 8 it declined. Injury at P28 provoked a different response, as shown in Figure 2D. One day after injury at P29 there was no change in ubiquitin in fraction 7 but a decline in fraction 8 was observed. Seven days after injury levels of the protein appeared to increase compared to age-matched controls in fraction 7 but declined in fraction 8. Such differences in ubiquitin levels in different fractions could explain why results obtained from whole extracts of the tissue (Figure 1) showed much less variation in response to injury. Therefore it seems that the overall changes in ubiquitin in response to injury can only be detected accurately using 2-D gel separation rather than a more standard SDS PAGE of tissue homogenates and are more likely to be due to modifications of the existing protein rather than its synthesis *de novo*. This is also indicated by the mostly non-significant changes in ubiquitin mRNA levels detected by qRT-PCR (Figure 1B).

### Cellular/Intracellular Distribution of Ubiquitin

**Figure 4:**
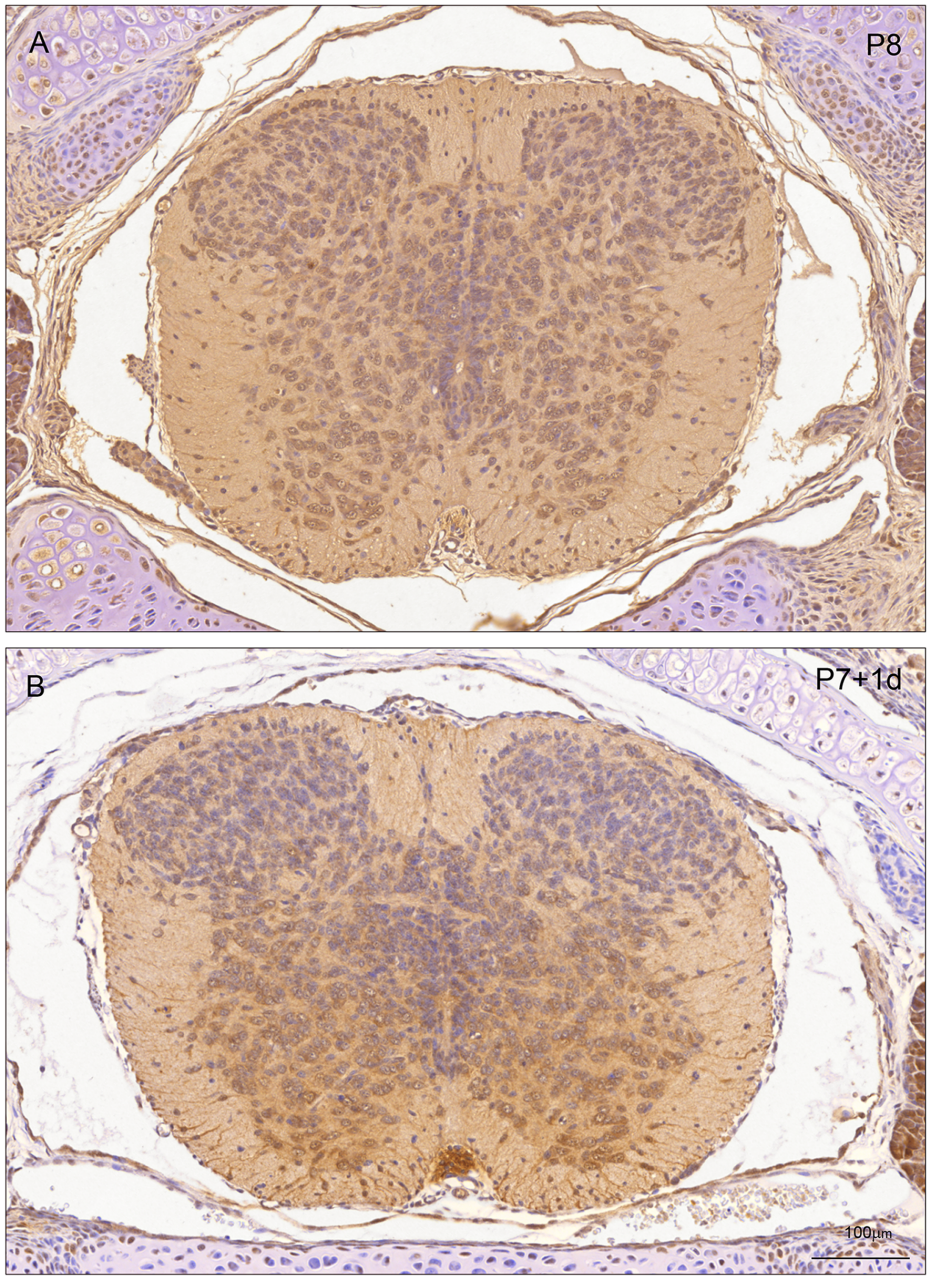
Immunocytochemical staining for ubiquitin in control (P8) and transected (P7+1 d) spinal cord of *Monodelphis domestica*. All sections are from matching levels of thoracic spinal cord rostral to the injury site. A: Eight days postnatal (P8) control. Note Immunostaining of well-developed motor neurons of the ventral horn grey matter, but also differentiated relay neurons of the intermediate grey including lateral horn sympathetic neurons. Early developing interneurons of the ‘head’ of the dorsal horn (*substantia gelatinosa* when differentiated) are weakly stained, in contrast to larger neuronal cells of Waldeyer surrounding the dorsal horn. B: P7+1 d post injury. Compared to the staining in the control (A) ubiquitin staining of the same level of the thoracic spinal cord demonstrates stronger staining in the ventral cord compared to the weakly reacting and unstained cells of the dorsal horn. A and B are of same magnification. Bar in B indicates 100 µm.

**Figure 5:**
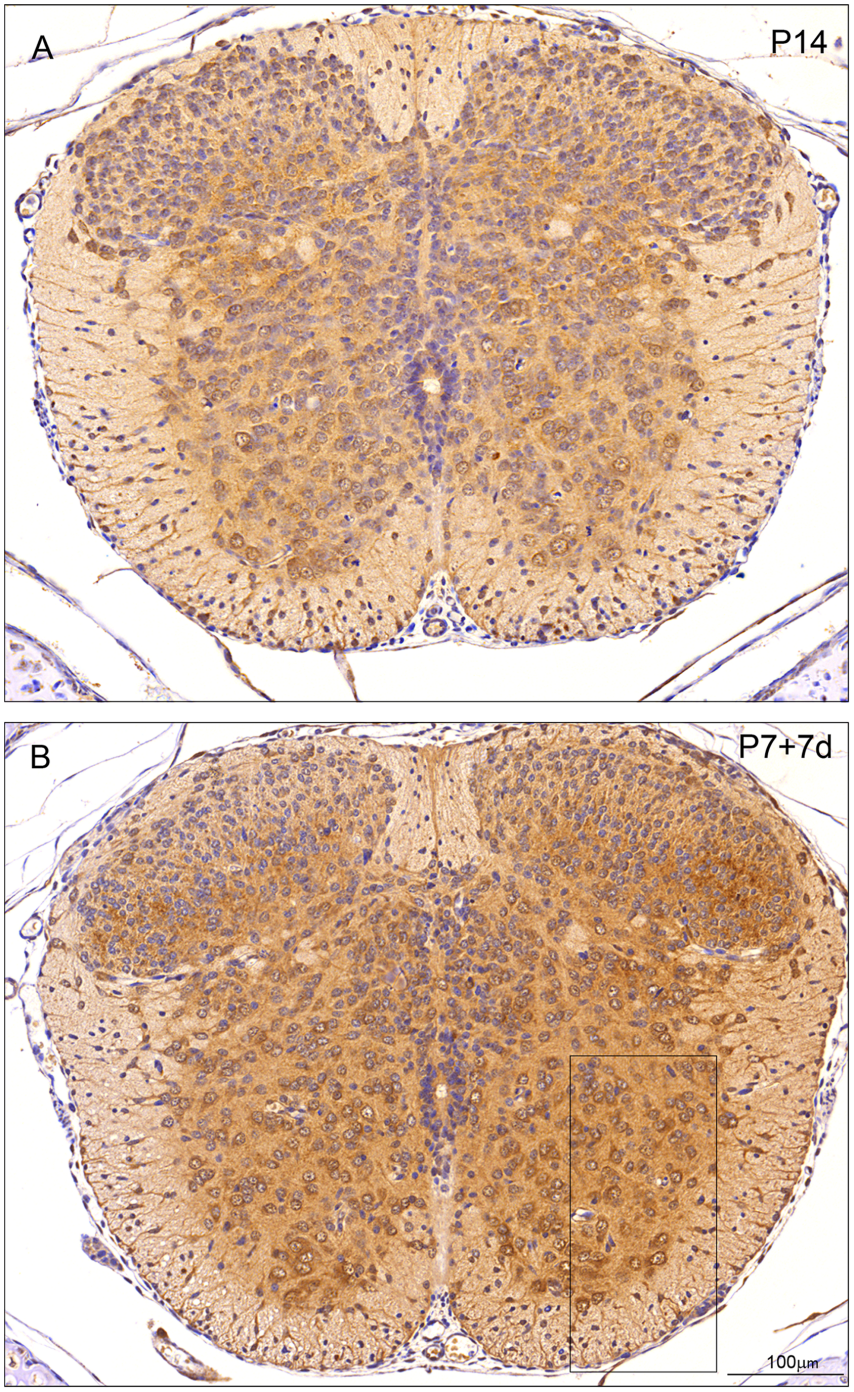
Immunocytochemical staining for ubiquitin in control (P14) and transected (P7+7 d) spinal cord of *Monodelphis domestica*. All sections are from matching levels of thoracic spinal cord rostral to the injury site. A: P14 control. Note strong staining of axial motor neurons in most ventral part of ventral horn. In white matter oligodendrocytes, radial glia and end feet are also strongly immunostained. B: P7+7 d post injury. Note strong staining of ventral horn interneurons and intermediate grey relay neurons. Interneurons in neck and ventral half of ‘head’ region of dorsal horn demonstrate highly increased ubiquitin immunoreactivity compared to P14 control (A) - especially in the neuropil. Endothelial cells and neuroependymal cells lining the central spinal cord are negative. Boxed area is shown in higher magnification in Figure 8A. A and B are of the same magnification. Bar in B indicates 100 µm.

**Figure 6:**
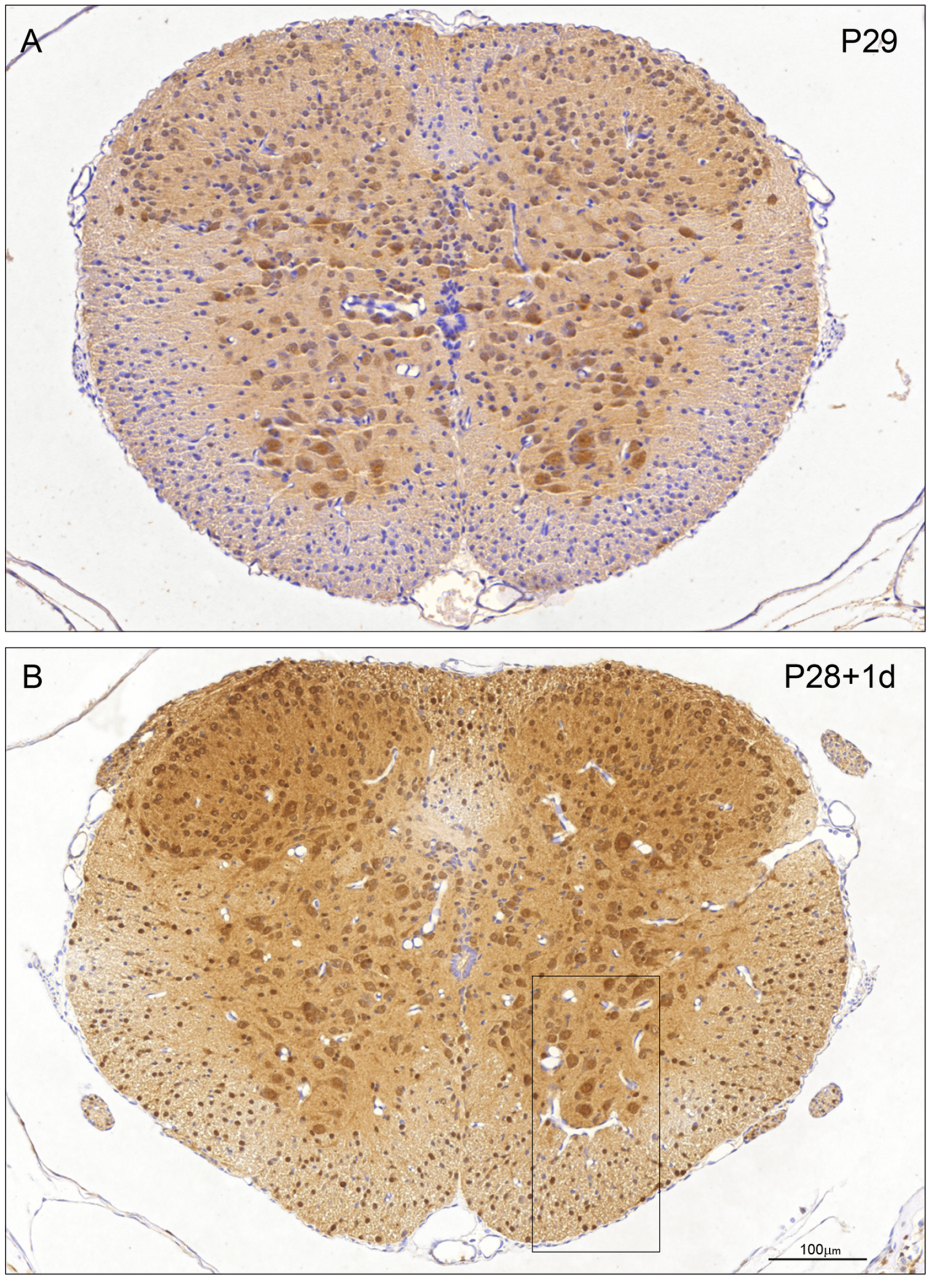
Immunocytochemical staining for ubiquitin in control (P29) and transected (P28+1 d) spinal cord of *Monodelphis domestica.* A: P29 (control). Note distribution of immunostaining for ubiquitin similar to P14 controls (Figure 5A) but immunostaining of large ventral horn motor neurons and neurons (cells of Waldeyer) surrounding dorsal horn is more prominent. B: P28+1 d post injury. Note strong immunostaining of ventral horn motor neurons as in P29 control (A). Also many more neurons in dorsal horn grey matter show prominent immunostaining and intense nuclear staining of oligodendroglia in the presumptive white matter and strong staining of glial processes including those of radial glia. These features in the boxed area are shown at higher power magnification in Figure 8 B. Endothelial cells and neuroependymal cells lining the central spinal cord are negative. A and B, same magnification. Bar in B: 100 µm.

**Figure 7:**
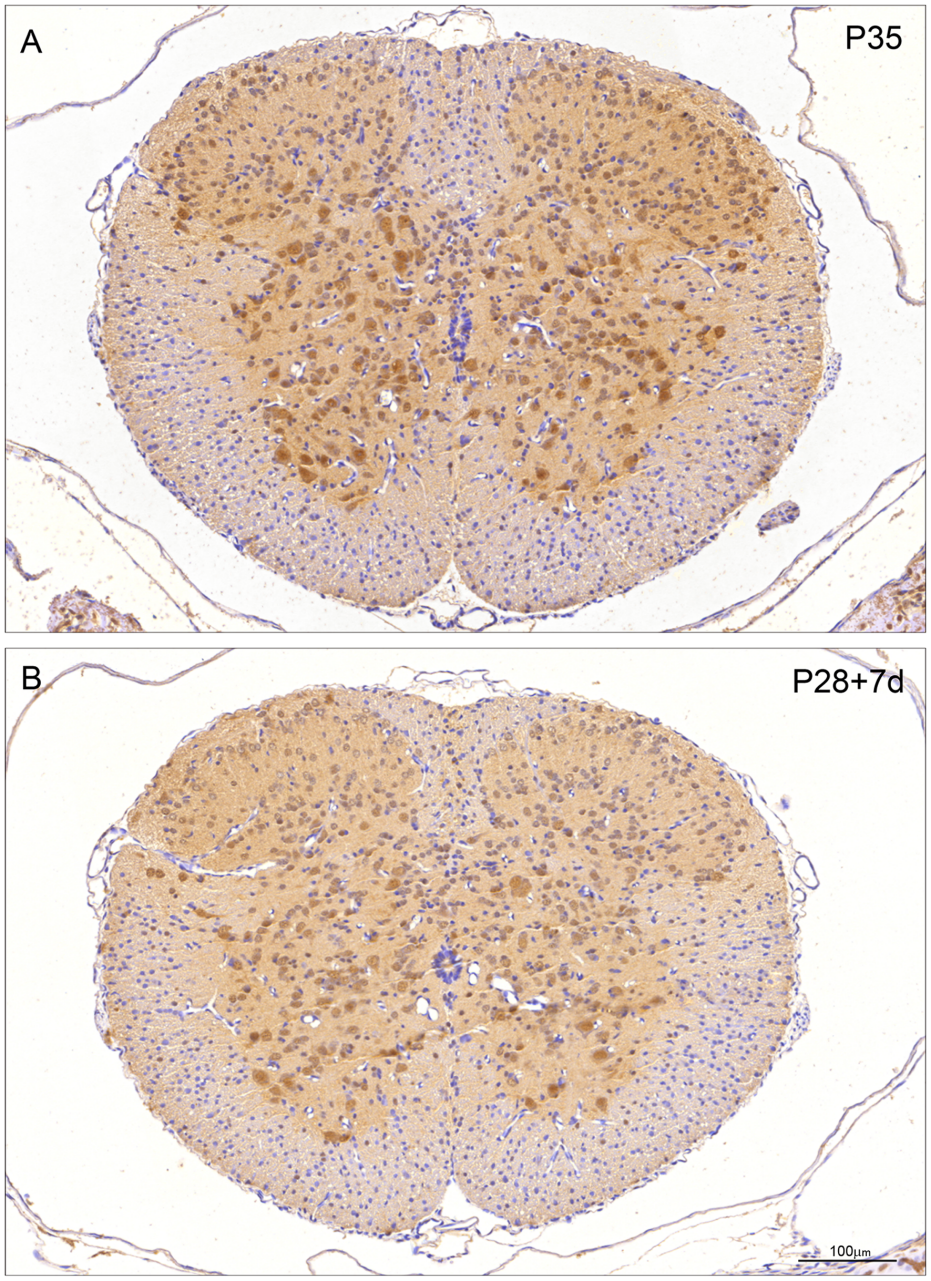
Immunocytochemical staining for ubiquitin in control (P35) and transected (P28+7 d) spinal cord of *Monodelphis domestica*. A: P35 control. Note ubiquitin staining similar to P29 (Figure 6A). Neuronal nuclei are as strongly stained as cytoplasm apart from Waldeyer cells at the tip of dorsal horn, which have stronger cytoplasmic staining. Some glial cells in white matter are positively stained. B: P28+7 d post injury. Note pattern of immunostaining is similar to that in age-equivalent control (P35, A) although less intense. Endothelial cells and neuroependymal cells lining the central spinal cord are negative. A and B, same magnification. Bar in B: 100 µm.

**Figure 8:**
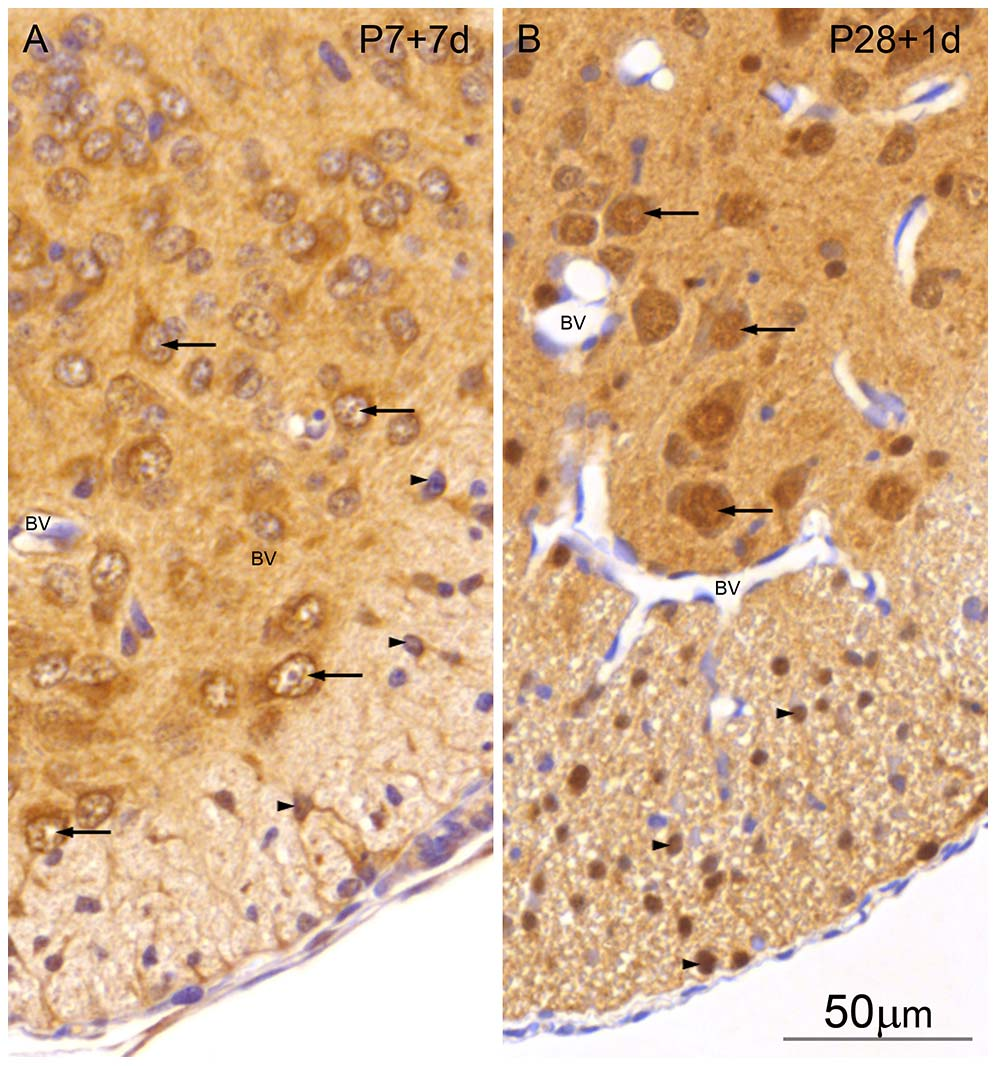
Higher power images of Immunocytochemical staining for ubiquitin in control and transected spinal cord of *Monodelphis domestica*. A: P7+7 d post injury. Lateral ventral section of spinal cord. Note large ventral horn neurons with strong cytoplasmic immunostaining for ubiquitin (arrows), and also cytoplasmic immunostaining of some oligodendroglia (arrow heads) and radial glia and processes. B: P28+1 day injury. Lateral ventral section of spinal cord. Note large ventral horn neurons with strong ubiquitin nuclear immunostaining for ubiquitin (arrows). Also prominent nuclear ubiquitin immunostaining of oligodendroglia (arrow heads) and radial glia and processes. There is no immunostaining of endothelial cells of cerebral blood vessels (bv) in either micrograph. A and B, same magnification. Bar in B: 50 µm.

Cellular distribution of ubiquitin was analyzed using immunocytochemical detection with cross-reacting antibodies to human ubiquitin [10]. Results are compared between age-matched control cords and those after spinal transection. Two to three sections from three separate cords at each age were used after careful matching of the spinal level from which they were selected. Results are illustrated in Figures 4–7 and under high magnification in Figure 8. A description of age related changes between P8 and P35 is given first, followed by the immunocytochemical changes identified at one and seven days after spinal transection at P7 or P28.

### Ubiquitin Immunostaining in Postnatal Monodelphis Thoracic Spinal Cord

Immunostaining for ubiquitin, in the grey matter of the thoracic spinal cord at P8 (Figure 4A) shows its immunoreactivity in well-developed motor neurons of the ventral horn but also in differentiated relay neurons of the intermediate grey including lateral horn sympathetic neurons. The early developing interneurons of the ‘head’ of the dorsal horn corresponding to *substantia gelatinosa* are weakly stained. This is in contrast to the larger neurons (cells of Waldeyer) surrounding the dorsal horn, which are rather more strongly stained. In the ventral and ventro-lateral white matter characteristic radial glial cell processes and end-feet as well as a few developing astrocytes are positively stained for ubiquitin. Very few oligodendrocytes are visible at this early age. The staining reaction product is mainly cytoplasmic and evenly distributed between neurons and glial cells and between differentiated (ventral horn) and differentiating (dorsal horn) neurons. Endothelial cells and ependymal cells of the central canal are not stained.

After a week of further development, at P14 (Figure 5A) axial motor neurons in the most ventral part of the ventral horn are now strongly stained in contrast to the more weakly stained ventral horn interneurons. Intermediate grey relay neurons and interneurons in the neck of the dorsal horn show moderate reactivity whereas neurons in the ‘head’ of the dorsal horn and cells of Waldeyer express a low level of ubiquitin reactivity similar to that at P8 (Figure 4A). The reactivity of radial glial processes and endfeet is now seen throughout the entire white matter, which now also exhibits strongly stained small oligodendrocytes.

After a further two weeks of development (at P29) the distribution of immunostaining for ubiquitin is similar to that in P14 controls, but the immunostaining of the large ventral horn motor neurons and the neurons (cells of Waldeyer) surrounding the dorsal horn is more prominent (Figure 6A). The staining of radial glia and small oligodendroglia is weaker than at P14. In addition a distinct shift of cellular distribution is now visible. Up to P14 ubiquitin immunoreactivity is predominantly cytoplasmic in both neurons and glial cells, but at P29 the majority of the neurons express strong nuclear and less cytoplasmic reactivity (Figure 6A).

At P35 ubiquitin staining (Figure 7A) is similar to that observed at P29 (Figure 6A). Neuronal nuclei are still as strongly stained as the cytoplasm apart from the Waldeyer cells at the tip of the dorsal horn, which exhibit a stronger cytoplasmic staining of the reaction product. A proportion of the glial cells in the white matter is also positively stained. As in the younger animals the endothelial cells and ependymal cells of the central canal are unstained.

### Ubiquitin Immunostaining in Postnatal Monodelphis Thoracic Spinal Cord Following Complete Transection

One day after injury at P7 (P7+1 d, Figure 4B), compared to its age-matched control (Figure 4A), ubiquitin staining of the same level of the thoracic spinal cord demonstrates a marked change in reactivity where the more developed ventral part is clearly more strongly stained compared to the weakly reacting and unstained cells of the dorsal horn.

Seven days after SCI at P7 (Figure 5B) there is a marked increase in ubiquitin reactivity in all neurons and glial cells compared to the P14 control (Figure 5A). Note in particular the strong staining of the ventral horn interneurons and of the intermediate grey relay neurons. Interneurons in the neck and ventral half of the ‘head’ region of the dorsal horn demonstrate highly increased ubiquitin immunoreactivity compared to the P14 control - especially in the neuropil. At higher magnification it can be seen that the ubiquitin staining in the ventral horn neurons is confined to the cytoplasm (Figure 8A, arrows) as is also the case for the radial glia and few oligodendroglia that are present (Figure 8A, arrowheads). As in control cords during development between P14 and P29, after injury a change in cellular distribution of ubiquitin is visible from predominantly cytoplasmic in both neurons and glial cells (illustrated for P7+7 d in Figure 8A) to mostly nuclear (Figures 6A and 8B). One day after injury at P28, the ventral horn motor neurons show prominent nuclear staining as in the P29 control cord (Figures 6B 8). However, there are two marked changes compared to the control. Firstly many more neurons in the dorsal horn grey matter show prominent immunostaining for ubiquitin. Secondly there is intense nuclear staining of the oligodendroglia in the presumptive white matter in addition to strong staining of glial processes including those of radial glia (Figure 8B). Seven days after injury at P28 (Figure 7B) the overall reaction pattern is similar to the P35 control specimens although in general the reaction product seems to be less intense.

## Discussion

### Ubiquitin Gene and Protein Expression Following Spinal Cord in Injury at P7 or P28

Previously we reported a differential response of ubiquitin to spinal cord injury, as identified by 2-D gel separation and validated using western blotting and qRT-PCR, in the segment of the developing opossum spinal cord caudal to the injury [10]. This was the first study, to our knowledge, that investigated expression of ubiquitin in response to spinal injury in an immature cord and in a marsupial. We have now extended this study to the segment of the cord rostral to the injury both during early development in *Monodelphis* and following a complete spinal transection. The results indicate that cellular distribution of ubiquitin and its expression change between the ages studied (P8 to P35) and differs depending on the age of injury (P7 or P28). We have identified that ubiquitin can be detected across the pH range of 6.48–7.64 as it was present in two fractions, Fraction 7 and 8 using the Off-gel Fractionator. There were some similarities and differences in ubiquitin expression levels between the segment of the cord caudal to the injury [10] and rostral to the injury (present study). Thus both caudal and rostral to the injury site, ubiquitin appeared to be reduced following injury at P7, although less so in the rostral segment. But following injury at P28 ubiquitin increased in the caudal segment [10] but appeared to decrease in the rostral segment (Figure 1A). The changes in ubiquitin protein in the caudal segment were matched by changes in expression [10], but not in the rostral segment (cf Figures 1A and 1B). Insofar as there were differences rostral and caudal to the lesion these most likely reflect the difference in the maturity of the cord at this age- previous work has demonstrated a very steep gradient of development in *Monodelphis* spinal cord [15].

Ubiquitin is a 76 amino acid or 8.5 kDa protein first identified in the thymus as a factor responsible for inducing differentiation of T and B cell via β-adrenergic receptors and adenylate cyclase activation [16]. It has been identified as a functional and well-conserved protein throughout 3 billion years of evolution from the yeast [17] to higher eukaryotes [17] but absent in Eubacteria and Archaea superkingdoms [18]. Ubiquitin plays an important role in the proteosomal degradation pathway where it is responsible for conjugating to other proteins targeted for degradation [19]. More recently it has been shown to play a role in cell signaling by competition with other ubiquitin-like molecules such as NEDD [20] and SUMO [21]–[23] because of their similarity in 3-dimensional structure and the enzymes that these molecules interact with such as the ubiquitin carboxyl-terminal hydrolase isozyme L3 (UCH-L3) [20] and E3-like ligases [22]–[23].

Earlier studies have identified several ubiquitin genes coding for mono- and poly-ubiquitin from the yeast, frog, mouse, chicken, cow and human [17], [24]–[29]. In addition, some of these ubiquitin genes also contain precursor sequences [17],[27]. One such fusion product was identified in the present study (heavier ubiquitin band in fractions 7 and 8, Figure 2). These ubiquitin fusion products can be cleaved as evidenced by the existence of an ubiquitin-specific processing protease [30]. Although these genes all code for the well-conserved mono-ubiquitin as their final protein product, it has been suggested that the different genes may play different roles in response to trauma through the cell’s heat shock response [24]–[25],[27]–[28]. Additionally, the poly-ubiquitin gene has been specifically implicated in developmentally programmed cell death [24]–[25],[27]–[28]. From the Monodelphis genome database, it is possible to predict at least two ubiquitin genes, a ribosomal protein S27a ubiquitin fusion product (NCBI RefSeq: XM_001375193.2 or Ensembl ID: ENSMODG00000001819) and a predicted poly-ubiquitin gene coding for tandem repeats of ubiquitin, Ubiquitin-C (NCBI RefSeq: XM_001366716.2 or Ensembl ID: ENSMODG00000003520). However, in the present study it was not possible to distinguish which ubiquitin was identified.

### Role of Ubiquitin in Neurological Disorders

Ubiquitin has been implicated in playing a role in several neurodegenerative diseases such as frontotemporal lobar degeneration [31]–[32], schizophrenia [33], Parkinson’s [34], spinal muscular atrophy [35], motor neuron disease/amyotrophic lateral sclerosis [36]–[40], often associated with filamentous inclusion bodies found in these types of disorder [31],[38]. In spinal cord injury ubiquitin has been detected in axonal swellings [41]; however in this study there was no evidence of ubiquitinated neuronal inclusions. Additionally, ubiquitin has been identified in reactive axonal swellings, which occur at both proximal and distal stumps of transected axons of the rat spinal cord [42]. Most of the studies suggest an association between ubiquitin and the proteosome degradation pathway that leads to diseases of the central nervous system and propose that it is its failure that is responsible for the progression of such diseases (reviewed by [43]–[44]).

### Role of Ubiquitin in Spinal Cord Development and Response to Injury

Little is known about the role of ubiquitin in the spinal cord during its development and in response to trauma, outside of its association with proteosome degradation pathway. Our previous [10] and present results show that ubiquitin may also be directly involved in processes taking part after spinal injury as its response to a complete mid thoracic transection was different at the two ages investigated. The two ages were chosen because we have demonstrated previously that if a complete transection at the mid thoracic level is performed at P7 in *Monodelphis*, these animals regenerate many of the axons that have been axotomised [4], re-grow substantial neuronal tissue across the injury site [5],[9] and recover a high degree of their locomotion when adult [9]. This stage of early CNS development was termed “permissive” to regeneration [9]–[10]. However if a similar injury is performed at P28, no re-growth of axons across the site of injury could be demonstrated, indicating that this stage of CNS development is “non permissive”, a situation that is similar to the adult [9]. In the present study we have shown that after spinal injury at P7, expression of ubiquitin in the segment of cord rostral to the injury site, as detected by qRT-PCR increased (but only statistically significantly seven days after injury, Figure 1) while its protein product levels in the whole homogenate barely changed. However if cord homogenate was first subjugated to a 2D separation and proteomic analysis, the results showed that changes in the isoelectric point of ubiquitin could be detected indicating a possible modification to the ubiquitin monomer (Figure 2). The apparent change in the size of ubiquitin protein peaks, as detected by the densitometry scans in both fractions, seems more likely to be due to modifications rather than to changes in its expression. The actual changes in total ubiquitin levels are indicated by western blot analysis of the whole tissue homogenate (Figure 1). The comparison indicates that to detect subtle changes in ubiquitin levels in response to injury, more accurate methods than 1-D PAGE separation are required.

### Changes in Cellular and Intracellular Distribution of Ubiquitin Following Injury in the Developing Spinal Cord

The results from western blotting (Figure 1A) gave an overall estimate of apparent changes in levels of ubiquitin during development and following injury. Results from immunocytochemical staining indicate changes in cellular and intracellular distribution, which do not always match the direction of the overall change. Thus there was a developmental increase in immunostaining for ubiquitin (Figures 4A, 5A, 6A) that matched an increase in protein level detected by western blotting (Figure 1A) However, following injury, immunostaining of some cellular populations increased markedly (Figure 4B, 5B, 6B) although the overall level of ubiquitin was reduced, albeit to a small extent (Figure 1A). This post injury increase was particularly distinct seven days post transection at P7, when a marked increase in ubiquitin staining was visible both in neurons and in glia (Figures 5B and 8). A similar immunocytochemical demonstration of ubiquitin cellular distribution was noted in the caudal spinal segment [10] but there was a difference in the levels of ubiquitin expression between the two segments: in the caudal segment expression did not change significantly, while in the rostral segment it increased one day after transection.

In contrast to injury at P7, ubiquitin expression after transection at P28 resulted in an apparent decrease one day after (not significant) or no change seven days later (Figure 1). The protein levels also seemed decreased in the whole homogenate but analysis of proteomic results revealed that most of this decrease was due to changes in fraction 8 (Figure 2D). This is in contrast to the results obtained from the same animals in the spinal cord segment caudal to the injury. In that segment ubiquitin levels in fraction 8 increased and its expression was also significantly elevated one day after injury [10]. Comparisons between levels of the protein and its mRNA transcript expression are difficult as it is not possible to use one as an indication of a change in expression for the other. In the present study this was further complicated by the possibility of the ubiquitin being the protein product of multiple genes possibly being expressed in *Monodelphis* genome under different conditions. However, based on the data for the protein, as identified by mass spectrometry and detected by the antibody used, the results show that the free ubiquitin monomer does appear to change its chemical properties and/or localization in development or in response to injury. As suggested before [10], these results indicate that in response to injury ubiquitin undergoes both synthesis *de novo* and post-translational modifications, as well as differential regulation seen by changes in isoelectric point, amount expressed and cellular localization.

The cellular distribution of ubiquitin also demonstrated an age-related shift from a more cytoplasmic immunostaining at an earlier age (up to P14) to a more nuclear distribution at later stages (P29–P35, see Figure 8 for examples). The nuclear immunostaining for ubiquitin was more prominent following injury. Glial cells were first seen to be clearly differentiated at P14, when immunostaining was present in radial glial cells and their endfeet, astrocytes and oligodendrocytes; this immunostaining became much more prominent by P28, particularly following injury.

### Mechanisms of Permissive and Non-permissive Axon Growth Following Spinal Cord Injury

In the past it was widely believed that axons in the adult CNS are unable to regenerate but this has been refuted by many studies over the past 30 years. The pioneering studies conducted by Aguayo and colleagues provided the evidence that neurons of the CNS retain their intrinsic ability to regenerate so long as the environment these neurons reside in is permissive such as within the peripheral nervous system [45]. This led to the conclusion that it is the CNS environment itself that was inhibiting central neuron regeneration [45]. Subsequent work led to the discovery of various molecules that inhibit neurite re-growth [46]–[49]. These molecules, associated with oligodendrocytic deposited myelin, [46]–[49] such as NOGO and OMgp were shown to be absent during development but were present in the mature CNS [50]. However, the results of spinal injury experiments in mice with gene deletions for NOGO, thought to be the principle myelin inhibitor, have been conflicting (see [51] for commentary). This together with genomic (rodent [52]; Monodelphis [5],[10]) and proteomic (rodents [53],[54] and cats [55]), studies of injured spinal cords demonstrated that a large number of genes and proteins show changes in expression following injury, suggesting that the limited response of the adult spinal cord to injury and the much more successful recovery following injury to the immature spinal cord is determined by much more complex mechanisms than the presence or absence of a single inhibitory factor. The present work suggests that ubiquitin and its pathway may well be involved as a component of the ability of the spinal cord to recover following trauma.

### Possible Pharmacological Intervention

The analysis of our proteomic and genomic data caudal to [10] and rostral to the injury site, indicates that ubiquitin could be a possible target for treating spinal cord injuries. The drug Bortezomib (Velcade, Millenium Pharmaceuticals) contains a boron atom that binds to the catalytic site of the 26S proteasome [56]. This interferes with the normal function of the proteasome in regulating protein expression and function by degradation of ubiquitinylated proteins. It has been approved for the treatment of multiple myeloma in the United States [57] and has been reported to reduce the size of ischaemic brain lesions [58]–[59]. Thus bortezomib treatment of older animals with spinal cord lesions in which ubiquitin expression is increased caudal to the injury site [10], might result in some preservation of neural tissue and improved neurological function following injury. However, the complexities of the changes in ubiquitin protein and expression with age, injury and cellular distribution suggests more will need to be known to make this a practicable approach.

### Conclusion

An important outcome of the present study is that understanding the molecular mechanisms of a particular gene or protein and its cellular localization is critical in determining the relevance and association to a particular disease. In this study we have not only shown the differences in transcripted mRNA but also the translation of its gene product, the ubiquitin protein. We have also shown the added complexity of the possibility of post-translational modification of ubiquitin suggesting the involvement of many other complex regulatory pathways. This is in addition to the discovery of several genes encoding mono-ubiquitin, poly-ubiquitin and ubiquitin fusion genes in the *Monodelphis domestica*. Both modifications and ubiquitin fusion products may possibly explain the differences in cellular localization throughout development observed in our study due to differences in cellular trafficking identified by different precursor tags attached to the ubiquitin sequence. This opens the possibility of a different approach to spinal cord injury by targeting ubiquitin.

### Limitations of the Study

#### Statistical analysis

In the present study no statistical analysis was performed for either the western blots or silver stained gels. Our aim was to identify possible changes in ubiquitin levels in response to spinal cord injury at two stages of development compared to their age-matched controls. The limitations of using silver staining as a visualization method for proteomics are well known. These include highly variable end staining of the gels thus making inter gel comparisons difficult and the narrow range of protein concentrations that can be detected accurately. Thus, densitometry analysis of such gels is not a quantitative method. Therefore, we have chosen to examine gross changes and possible trends as opposed to actual values and only note differences that occur within one gel. To ensure this technical aspect was replicated in a duplicate gel, all samples were run twice. We also confirmed results from silver stained gels with western blotting and demonstrated that differences observed from densitometry scans correspond with those obtained using antibodies.

Off-gel fractionation was performed on one pooled sample for each age group. Cords were pooled from several animals, particularly at younger ages, to obtain enough material. In addition pooling has the advantage that it gives an average estimate from many individuals avoiding the problem that might arise with variability of single samples from individual animals. The whole proteomic separation was carried out on one pooled sample because to obtain a biological replication would have required a very large number of additional pups, which we considered would not have been ethically justifiable. However we did collect pups from different litters for every method we used and made replicate blots of both whole homogenates and 1D LDS gels. We have validated the biological reproducibility of the proteomic analysis on several independent samples of P35 cords as we reported in the previously published paper [10].

#### Ubiquitin gene annotation

The published *Monodelphis* genome is not well annotated with the identification of many proteins/genes still being based on sequence homology with other species and labeled as “predicted”. It is not known how many poly ubiquitin genes there are. Based on predicted sequences annotated in the database we could identify at least two poly-ubiquitin genes. The PCR primers used in our studies were specific to one of the ubiquitin genes (XM_001375193). Gel electrophoresis on agarose showed amplification of a single PCR product. With the possibility of multiple splice variants of the gene that the PCR primer was based on as well as mRNA splice variants, further analysis by Southern (gene identification) and northern (mRNA) blot would be required. At present, the aim of the study was to confirm and extend our earlier observation that ubiquitin changes its expression and cellular localisation both in development and in response to spinal injury in the rostral spinal cord, in addition to previously published results for the caudal segment [10].

#### Cellular localization

The antibodies to ubiquitin that we used were raised against the human protein. They are known to cross react with rat ubiquitin, which we used previously to validate it for cross reactivity with the *Monodelphis* antigen [10]. However, the antibody only picks up the lower band of ubiquitin visible on 1D gels (see results of western blots). It is therefore possible that we may have missed some additional changes in ubiquitin cellular localization in development and response to injury. This would require *Monodelphis*-specific antibodies, which are not available at present.

## References

[1] Ramon Y, Cajal S (1913) Degeneration and regeneration of the nervous system. London: Oxford Univ Press.

[2] Saunders N, Dziegielewska K (2000) Recovery from injury in the immature mammalian spinal cord. In: Saunders NR, Dziegielewska KM, editors Degeneration and regeneration in the nervous system Amsterdam: Harwood Academic Publishers: 17–52.

[3] Terman J, Wang X, Martin G (2000) Repair of the transected spinal cord at different stages of development in the North American opossum, Didelphis virgiana. Brain Res Bull 53: 845–855. 11179852 10.1016/s0361-9230(00)00431-7

[4] Fry E, Stolp H, Lane M, Dziegielewska K, Saunders N (2003) Regeneration of supraspinal axons after complete transection of the thoracic spinal cord in neonatal opossums (*Monodelphis domestica*). J Comp Neurol 466: 422–444. 14556298 10.1002/cne.10904

[5] Lane M, Truettner J, Brunschwig J, Gomez A, Bunge M, et al. (2007) Age-related differences in the local cellular and molecular responses to injury in developing spinal cord of the opossum, Monodelphis domestica. Eur J Neurosci 25. 10.1111/j.1460-9568.2007.05439.x 17432961

[6] Fry E, Saunders N (2000) Spinal repair in immature animals: A novel approach using the South American opossum *Monodelphis domestica* . Clin Exp Pharm Physio 27: 542–547. 10.1046/j.1440-1681.2000.03296.x 10874515

[7] Saunders N, Adam E, Reader M, Møllgård K (1989) Monodelphis domestica (grey short-tailed opossum): an accessible model for studies of early neocortical development. Anat Embryol (Berl) 180: 227–236. 2596703 10.1007/BF00315881

[8] Mikkelsen TS, Wakefield MJ, Aken B, Amemiya CT, Chang JL, et al (2007) Genome of the marsupial Monodelphis domestica reveals innovation in non-coding sequences. Nature 447: 167–177. 17495919 10.1038/nature05805

[9] Wheaton B, Callaway J, Ek CJ, Dziegielewska KM, Saunders NR (2011) Spontaneous development of full weight-supported stepping after complete spinal cord transection in the neonatal opossum, *Monodelphis domestica* . PLoS One 6: e26826. 22073202 10.1371/journal.pone.0026826 PMC3206848

[10] Noor NM, Steer DL, Wheaton B, Ek C, Truettner J, et al (2011) Age-dependent changes in the proteome following complete spinal cord transection in a postnatal South American opossum (*Monodelphis domestica*). PLoS One 6: e27465. 22110655 10.1371/journal.pone.0027465 PMC3217969

[11] Buckley S, Aranda-Orgilles B, Strikoudis A, Apostolou E, Loizou E, et al (2012) Regulation of pluripotency and cellular reprogramming by the ubiquitin-proteosome system. Cell Stem Cell 11: 1–16.22770234

[12] Bradford M (1976) A rapid and sensitive method for the quantification of microgram quantities of protein utilizing the principle of protein-dye binding. Analytical Biochem 72: 248–254. 10.1016/0003-2697(76)90527-3 942051

[13] Baker R, Board P (1991) The human ubiquitin-52 amino acid fusion protein gene shares several structural features with mammalian ribosomal protein gene. Nucleic Acids Res 19: 1035–1040. 1850507 10.1093/nar/19.5.1035 PMC333777

[14] Mezquita J, Pau M, Mezquita C (1997) Characterization and expression of two chicken cDNAs encoding ubiquitin fused to ribosomal proteins of 52 and 80 amino acids. Gene 195: 313–319. 9305777 10.1016/s0378-1119(97)00189-3

[15] Møllgård K, Balslev Y, Janas M, Treherne J, Saunders N, et al (1994) Development of spinal cord in the isolated CNS of the neonatal mammal (the opossum *Monodelphis domestica*) maintained in longterm culture. J Neurocytol 23: 151–165. 8006676 10.1007/BF01181557

[16] Goldstein G, Scheid M, Hammerling U, Boyse E, Schlesinger D, et al (1975) Isolation of a polypeptide that has lymphocyte-differentiating properties and is probably represented universally in living cells. Proc Nat Acad Sci USA 72: 11–15. 1078892 10.1073/pnas.72.1.11 PMC432229

[17] Ozkaynak E, Finley D, Varshavsky A (1984) The yeast ubiquitin gene: head-to-tail repeats encoding a polyubiquitin precursor protein. Nature 312: 663–666. 6095120 10.1038/312663a0

[18] Hochtrasser M (2009) Origin and function of ubiquitin-like proteins. Nature 458: 422–429. 19325621 10.1038/nature07958 PMC2819001

[19] Ebrahimi-Fakhari D, Cantuti-Castelvetri I, Fan Z, Rockenstein E, Masliah E, et al (2011) Distinct roles *in vivo* for the ubiquitin-proteasome system and the autophagy-lysosomal pathway in the degradation of α-Synuclein. J Neuroscience 31: 14508–14520. 10.1523/JNEUROSCI.1560-11.2011 PMC3587176 21994367

[20] Wada H, Kito K, Caskey L, Yeh E, Kamitani T (1998) Cleavage of the C-terminus of NEDD8 by UCH-L3. Biochem Biophys Res Commun 25: 688–692. 10.1006/bbrc.1998.9532 9790970

[21] Johnson E, Gupta A (2001) An E3-like factor that promotes SUMO conjugation to the yeast septins. Cell 106: 713–718. 10.1016/s0092-8674(01)00491-3 11572779

[22] Nishida T, Yasuda H (2002) PIAS1 abd PIASxα function as SUMO-E3 ligases toward androgen receptor and repress androgen receptor-dependent transcription. J Biol Chem 277: 41311–41317. 12177000 10.1074/jbc.M206741200

[23] Sachdev S, Bruhn L, Sieber H, Pichler A, Melchior F, et al (2001) PIASy, a nuclear matrix-associated SUMO E3 ligase, represses LEF1 activity by sequestration into nuclear bodies. Genes Dev 15: 3088–3103. 11731474 10.1101/gad.944801 PMC312834

[24] Bond U, Schlesinger M (1985) Ubiquitin is a heat shock protein in chicken embryo fibroblasts. Mol and Cell Biol 5: 949–956. 2987683 10.1128/mcb.5.5.949-956.1985 PMC366809

[25] Bond U, Schlesinger M (1986) The chicken ubiquitin gene contains a heat-shock promoter and expresses an unstable mRNA in heat-shocked cells. Mol and Cell Biol 6: 4602–4610. 3025663 10.1128/mcb.6.12.4602-4610.1986 PMC367245

[26] Dworkin-Rastl E, Shrutkowski A, Dworkin M (1984) Multiple ubiquitin mRNAs during Xenopus laevis development contain tandem repeats of the 76 amino acid sequence. Cell 39: 321–325. 6209017 10.1016/0092-8674(84)90010-2

[27] Ozkaynak E, Finley D, Solomon M, Varshavsky A (1987) The yeast ubiquitin genes: a family of natural gene fusions. The EMBO Journal 6: 1429–1439. 3038523 10.1002/j.1460-2075.1987.tb02384.x PMC553949

[28] Schwartz L, Myer A, Kosz L, Engelstein M, Maier C (1990) Activation of polyubiquitin gene expression during developmentally programmed cell death. Neuron 5: 411–419. 2169771 10.1016/0896-6273(90)90080-y

[29] Wiborg O, Pedersen M, Wind A, Berglund L, Marcker K, et al (1985) The human ubiquitin multigene family: some genes contain multiple directly repeated ubiquitin coding sequences. The EMBO Journal 4: 755–759. 2988935 10.1002/j.1460-2075.1985.tb03693.x PMC554252

[30] Bachmair A, Finley D, Varshavsky A (1986) *In vivo* half-life of a protein is a function of its amino-terminal residue. Science 234: 179–186. 3018930 10.1126/science.3018930

[31] Forno L, Langston J, Herrick M, Wilson J, Murayama S (2002) Ubiquitin-positive neuronal and tau 2-positive glial inclusions in frontotemporal dementia of motor neuron type. Acta Neuropathol 103: 599–606. 12012092 10.1007/s00401-001-0509-1

[32] Josephs K, Holton J, Rossort M, Godbolt A, Ozawa T, et al (2004) Frototemporal lobar degeneration and ubiquitin immunohistochemistry. Neuropathol and Applied Neurobiol 30: 369–373. 10.1111/j.1365-2990.2003.00545.x 15305982

[33] Altar C, Jurata L, Charles V, Lemire A, Liu P, et al (2005) Deficient hippocampal neuron expression of proteosome, ubiquitin and mitochondrial genes in multiple schizophrenia cohorts. Biol Psychiatry 58: 85–96. 16038679 10.1016/j.biopsych.2005.03.031

[34] Gai W, Blessing W, Blumbergs P (1995) Ubiquitin-positive degenerating neurites in the brainstem in Parkinson’s disease. Brain 118: 1447–1459. 8595476 10.1093/brain/118.6.1447

[35] Hiraga T, Leipold H, Cash W, Troyer D (1993) Reduced numbers and intense anti-ubiquitin immunostaining of bovine motor neurons affected with spinal muscular atrophy. J Neurol Sciences 118: 43–47. 10.1016/0022-510x(93)90244-s 7693876

[36] Leigh P, Whitwell H, Garofalo O, Buller J, Swash M, et al (1991) Ubiquitin-immunoreactive intraneuronal inclusions in amyotrophic lateral sclerosis. Brain 114: 775–788. 1646064 10.1093/brain/114.2.775

[37] Matsumoto S, Goto S, Hirofumi K, Imai T, Murakami N, et al (1993) Ubiquitin-positive inclusion in the anterior horn cells in subgroups of motor neuron diseases: a comparative study of adult-onset amyotrophic lateral sclerosis, juvenile amyotrophic lateral sclerosis and Werdnig-Hoffman disease. J Neurol Sciences 115: 208–213. 10.1016/0022-510x(93)90226-o 8387100

[38] Matsumoto S, Hirano A, Goto S (1990) Ubiquitin-immunoreactive filamentous inclusions in anterior horn cells of Guamanian and non-Guamanian amyotrophic lateral sclerosis. Acta Neuropathol 80: 233–238. 2169172 10.1007/BF00294639

[39] Okamoto K, Murakami N, Kusaka H, Yoshida M, Hashizume Y, et al (1992) Ubiquitin-positive intraneuronal inclusions in the extramotor cortices of presenile dementia patients with motor neuron disease. J Neurol 239: 426–430. 1333007 10.1007/BF00856806

[40] Schiffer D, Autillo-Gambetti L, Chio A, Gambetti P, Giordana M, et al (1991) Ubiquitin in motor neuron disease: Study at the light and electron microscope. J Neuropathol and Expt Neurol 50: 463–473. 10.1097/00005072-199107000-00007 1648124

[41] Martin J, Mather K, Swash M, Garofalo O, Dale G, et al (1990) Spinal cord trauma in man: Studies of phosphorylated neurofilament and ubiquitin expression. Brain 113: 1553–1562. 1700924 10.1093/brain/113.5.1553

[42] Li G, Farooque M (1996) Expression of ubiquitin-like immunoreactivity in axons after compression trauma to rat spinal cord. Acta Neuropathol 91: 155–160. 8787148 10.1007/s004010050407

[43] Ardley H, Hung C, Robinson P (2005) The aggravating role of the ubiqutiin-proteasome system in neurodegeneration. FEBS Letters 579: 571–576. 15670810 10.1016/j.febslet.2004.12.058

[44] Ciechanover A, Brundin P (2003) The ubiquitin proteasome system in neurodegenerative diseases: sometimes the chicken, sometimes the egg. Neuron 40: 427–446. 14556719 10.1016/s0896-6273(03)00606-8

[45] Richardson P, McGuinness U, Aguayo A (1980) Axons from CNS neurones regenerate into PNS grafts. Nature 284: 264–265. 7360259 10.1038/284264a0

[46] Craveiro L, Hakkoum D, Weinmann O, Montani L, Stoppini L, et al (2008) Neutralization of the membrane protein Nogo-A enhances growth and reactive sprouting in established organotypic hippocampal slice cultures. Eur J Neurosci 28: 1808–1824. 18973596 10.1111/j.1460-9568.2008.06473.x

[47] Ji B, Case L, Liu K, Shao Z, Lee X, et al (2008) Assessment of functional recovery and axonal sprouting in oligodendrocyte-myelin glycoprotein (OMgp) null mice after spinal cord injury. Mol Cell Neurosci 39: 258–267. 18692574 10.1016/j.mcn.2008.07.004 PMC2646371

[48] Su Y, Wang F, Zhao SG, Pan SH, Liu P, et al (2008) Axonal regeneration after optic nerve crush in Nogo-A/B/C knockout mice. Mol Vis 14: 268–273. 18334965 PMC2263011

[49] Tomita K, Kubo T, Matsuda K, Yano K, Tohyama M, et al (2007) Myelin-associated glycoprotein reduces axonal branching and enhances functional recovery after sciatic nerve transection in rats. Glia 55: 1498–1507. 17705198 10.1002/glia.20566

[50] Huber A, Weinmann O, Brosamle C, Oertle T, Schwab M (2002) Patterns of Nogo mRNA and protein expression in the developing and adult rat after CNS lesions. J Neurosci 22: 3553–3567. 11978832 10.1523/JNEUROSCI.22-09-03553.2002 PMC6758364

[51] Silver J (2010) Much ado about Nogo. Neuron 66: 619–621. 20547119 10.1016/j.neuron.2010.05.028

[52] Aimone JB, Leasure JL, Perreau VM, Thallmair M (2004) Spatial and temporal gene expression profiling of the contused rat spinal cord. Exp Neurol 189: 204–221. 15380473 10.1016/j.expneurol.2004.05.042

[53] Kunz S, Tegeder I, Coste O, Marian C, Pfenninger A, et al (2005) Comparative proteomic analysis of the rat spinal cord in inflammatory and neuropathic pain models. Neurosci Lett 381: 289–293. 15896486 10.1016/j.neulet.2005.02.022

[54] Yan X, Liu J, Luo Z, Ding Q, Mao X, et al (2010) Proteomic profiling of proteins in rat spinal cord induced by contusion injury. Neurochemistry International 56: 971–983. 20399821 10.1016/j.neuint.2010.04.007

[55] Van den Bergh G, Clerens S, Firestein B, Burnat K, Arckens L (2006) Development and plasticity-related changes in the protein expression patterns in cat visual cortex: a flurorescent two-dimensional difference gel electrophoresis approach. Proteomics 6: 3821–3832. 16739136 10.1002/pmic.200500570

[56] Bonvini P, Zorzi E, Basso G, Rosolen A (2007) Bortezomib-mediated 26S proteasome inhibition causes cell-cycle arrest and induces apoptosis in CD-30+ anaplastic large cell lymphoma. Leukemia 21: 838–842. 17268529 10.1038/sj.leu.2404528

[57] Takimoto C, Calvo E (2008) Principles of Oncologic Pharmacotherapy. Ch 3 Appendix 3, in Pazdur R, Wagman LD, Camphausen KA, Hoskins WJ (Eds) Cancer Management: A Multidisciplinary Approach, 11th edition. Available: http://www.cancernetwork.com/cancermanagement-11/.

[58] Henninger N, Sicard K, Bouley J, Fisher M, Stagliano N (2006) The proteasome inhibitor VELCADE ® reduced infarction in rat models of focal cerebral ischemia. Neurosci Lett 398: 300–305. 16490315 10.1016/j.neulet.2006.01.015

[59] Zhang L, Zhang Z, Liu X, Hozeska A, Stagliano N, et al (2006) Treatment of embolic stroke in rats with bortezomib and recombinant human tissue plasminogen activator. Thromb Haemost 95: 166–173. 16543976

